# Suck, swallow and breathing coordination in infants with infantile colic

**DOI:** 10.4102/sajcd.v62i1.115

**Published:** 2015-12-02

**Authors:** Hanlie Degenaar, Alta Kritzinger

**Affiliations:** 1 Institute of Psychology and Wellbeing, North-West University, Potchefstroom Campus, South Africa; 2 Department of Speech-language Pathology and Audiology, University of Pretoria, South Africa

## Abstract

**Background:**

There appears to be a perception amongst parents and in popular literature that infantile colic is caused by feeding difficulties. Limited support for this perception is found in scientific literature. Whilst there is scientific evidence that suck, swallow and breathing are key components of successful feeding, these components and the coordination thereof in infants with colic have not been extensively researched.

**Objective:**

The objective of the study was to explore the suck, swallow and breathing coordination in infants with infantile colic and compare it with infants without the condition.

**Method:**

An assessment protocol for suck, swallow and breathing coordination was compiled from literature. This protocol was performed on a research group of 50 infants, independently diagnosed with infantile colic, and a control group of 28 infants without the condition. All participants were from two rural towns in the North–West province, South Africa, selected with a snowball selection method and strict selection criteria. The study followed a static comparison group design.

**Results:**

A significant difference in the key components of feeding and the presence of colic in participants of four age categories were found. The correlation between postural control and the presence of infantile colic were sustained in participants from 2–19 weeks old.

**Conclusion:**

Suck, swallow and breathing were found to be significantly associated with infantile colic. The findings should be investigated further. It appears that speech-language therapists may play an expanding role in infantile colic.

## Introduction

Infantile colic is a condition that commonly occurs in 10%–40% of typical, healthy and growing infants whether they are breastfeeding or bottle feeding (Deshpande, 2003; Kheir, 2012; Søndergaard, Skajaa & Henriksen, 2000) but lasts only until the age of four months (Cohen-Silver & Ratnapalan, 2009; Kheir, 2012; Savino, 2007). The description of infantile colic mostly used in literature is still based on the definition of Wessel, Cobb, Jackson, Harris and Detwiler (1954). The condition is described as sudden onset periods of high-pitched crying without an explainable cause (Kheir, 2012), exceeding three hours per day in duration (Deshpande, 2003; Gudmundsson, 2010) and lasting for more than three days within a period of three weeks (Lucassen *et al.*, 2001; Savino, 2007).

Several factors have already been identified that may increase the risk of infantile colic. These include gastro-oesophageal reflux (Heine, 2006), increased levels of gastrointestinal hormones (Savino *et al.*, 2006), flora (Savino *et al.*, 2010), esophagitis (Berezin, Glassman, Bostwick & Halata, 1995), low birth weight (Søndergaard *et al.*, 2000), maternal smoking (Reijneveld, Brugman & Hirasing, 2000), lactose intolerance (Kanabar, Randhawa & Clayton, 2001) and feeding difficulties (Gudmundsson, 2010; Miller-Loncar, Bigsby, High, Wallach & Lester, 2004). The etiology appears to be unknown (Kheir, 2012; Lucassen *et al.*, 2001) and no standard treatment protocol for infantile colic has been indicated (Hall, Chesters & Robinson, 2012). Despite limited clinical evidence that feeding problems occur in infants with infantile colic (Miller-Loncar *et al.*, 2004) the perception amongst the general public and the popular literature are that difficulties with sucking and swallowing causes colic (Bailey, D’ Auria & Haushalter, 2012). Advertisements and articles in baby magazines reinforce the perception that colic can be alleviated by certain bottles, teats and a change in handling the infant (Catherine, Ko & Barr, 2008). This difference in the common perception of infantile colic and clinical evidence has not been investigated.

Successful feeding is determined by three factors, namely the infant’s oral-motor feeding movements, the sucking, swallowing and breathing coordination (SSBC) and the interaction during feeding with the caregiver (Hall, 2001; Morris & Klein, 2001; Swigert, 2009). It is generally accepted that infants with infantile colic have normal sucking and swallowing skills, adequate growth and adequate nutrition (Deshpande, 2003; Lucassen, 2010). Miller-Loncar *et al.* (2004) however found that infants with infantile colic had less rhythmic sucking, organised feeding behaviour and interactive responses during feeding than a control group. There is also evidence that the condition negatively impacts on infant-caregiver interaction (Brown, Thoyre, Pridham & Schubert, 2009; Miller-Loncar *et al.*, 2004; Rossetti, 2001) and adds to parental frustration (Deshpande, 2003; Hall *et al.*, 2012), postnatal depression (Vik *et al.*, 2009), family stress (Beebe, Casey & Pinto-Martin, 1993) and family conflict (Raiha, Lehtonen, Korhonen & Korvenranta, 1997). Based on these results there may be a relationship between infantile colic, SSBC and caregiver interaction. The feeding process, in particular SSBC, has not yet been investigated in infants with infantile colic.

Successful feeding in an infant younger than four months depends on a well-developed SSBC pattern (Arvedson & Brodsky, 2002; Wolf & Glass, 1992). At four months most neonatal reflexes, the Moro, rooting, sucking, tonic neck reflex and palmar grasp, disappear (Alexander, Boehme & Cupps, 1993; Morris & Klein, 2001) and successful feeding is less dependent on SSBC. The integration of reflexes into typical movement patterns may explain why infantile colic eases or disappears at age four months (Savino & Tarasco, 2010) and also strengthens the idea that SSBC may play a role in the condition.

SSBC is a fundamental sensory motor pattern which organises the infant’s neuro-motor behaviour (Oetter, Richter & Frick, 1995) and is present since birth in typical full-term infants (Swigert, 2009). SSBC is considered as the first development pattern that involves successive, timed and sequenced movement of different structures (Barlow, 2009) with a significant influence on the infant’s postural control, psychosocial development and emotional state (Brown *et al.*, 2009; Oetter *et al.*, 1995). That is because SSBC involves various bony structures, muscles, cervical and cranial nerves (Barlow, 2009; Seikel, Douglas & Drumright, 2010) and is also linked to the limbic system, reticular formation and autonomic nervous system (Oetter *et al.*, 1995; Wolf & Glass, 1992). A disturbance in SSBC could by implication disturb the infant’s sleep patterns, alertness, attention and sensory threshold (Blanche, Botticelli & Hallway 1995; Hemmi, Wolke & Schneider, 2011; Oetter *et al.*, 1995).

**FIGURE 1:**
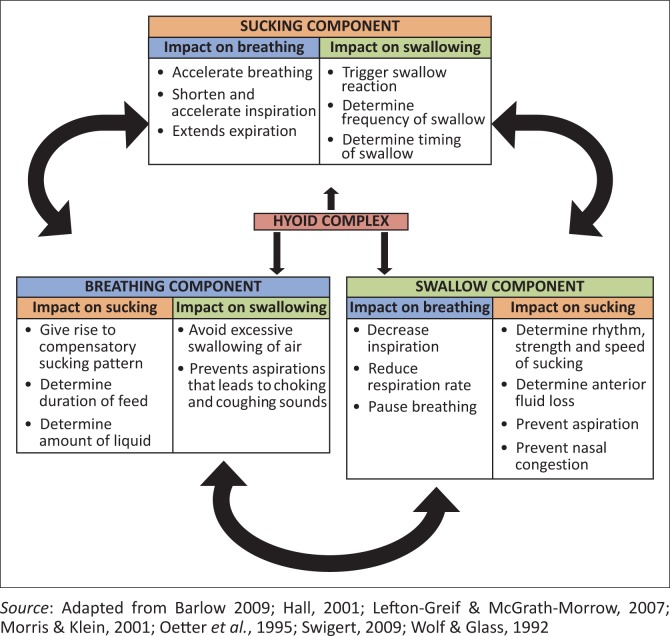
The relationship between the different components of SSAK.

SSBC is a complex, synchronised movement pattern for feeding in infants and involves three functional components synchronised by the hyoid complex. Figure 1 displays the relationship between the three components of SSBC.

As indicated in Figure 1 the hyoid bone and the muscles attached to the structure are central to SSBC. The suprahyoid and infrahyoid muscles stabilise the hyoid bone which should be aligned with other bony structures involved in SSBC to achieve effective sequential movements (Morris & Klein, 2001; Perkins & Kent, 1986; Wolf & Glass, 1992). The hyoid bone provides coordination of muscle movement around the bony structures involved in sucking, swallowing and breathing (Oetter *et al.*, 1995, Perkins & Kent, 1986; Seikel *et al.*, 2010). Any disturbance of the hyoid complex will disturb SSBC and a slight disturbance in SSBC may lead to a slight disturbance in the infant’s feeding process. The conclusion is that a subtle disturbance in the balance between the components of SSBC may cause a number of feeding difficulties in young infants. Clinical observable factors could assist to identify and assess a SSBC disturbance.

**FIGURE 2:**
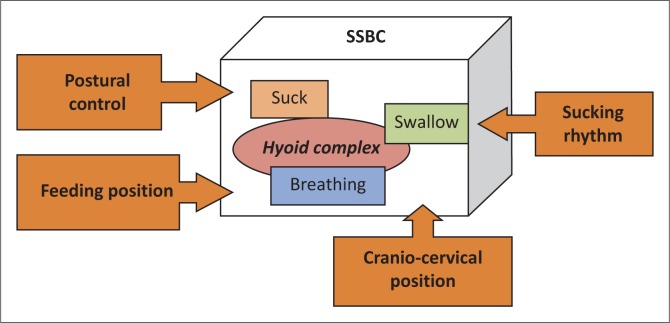
Observable factors involved in disturbance of sucking, swallowing and breathing coordination.

The diagnosis of infantile colic is currently characterised by parental perception of the infant’s behaviour and the elimination of other medical conditions (Deshpande, 2003; Kanabar, 2008; Savino & Tarasco, 2010) without reference to the feeding process. Parents base their perception of colic on the acoustic characteristic of the infant’s cry and behaviour of fisting, flatulence and pulling legs towards the abdomen (Deshpande, 2003; Lester, Boukydis, Garcia-Coll, Hole & Peucker, 1992; Savino, 2007; St James-Roberts, Conroy & Wilsher, 1996). Parental descriptions of colic vary as perceptions are determined by socio-economic status, education, religion, previous experience of an infant with infantile colic, environmental factors, personality, parental age, marital status and the presence of a support system (Rossetti, 2001). Differences in descriptions are therefore not objective and reliable for assessment of the condition. Observation of factors that may disturb SSBC may contribute to objectivity in the assessment of infants with infantile colic. Postural control, feeding position, sucking rhythm and cranio-cervical position are four observable factors that determine the effective functioning of the hyoid complex and the ultimately SSBC (Barlow, 2009; Oetter *et al.*, 1995; Wolf & Glass, 1992). The observable factors that influence SSBC are depicted in Figure 2.

According to Figure 2, *postural control* is the ability to align bony structures and maintain alignment during an activity (Cupps, 1997) and is therefore essential for feeding (Arvedson & Lefton-Greif, 1996; Hall, 2001; Morris & Klein, 2001; Rogers, 1996). Poor alignment leads to less efficient feeding, increased energy expenditure, limited endurance and prolonged duration of feeding (Hall, 2001; Morris & Klein, 2001; Wolf & Glass, 1992). *Feeding position* is important since any external force on the hyoid complex or bony structures involved in breathing may disturb SSBC (Morris & Klein, 2001; Oetter *et al.*, 1995; Perkins & Kent, 1986). Several authors have stressed the importance of the feeding position for infants (Arvedson & Lefton-Greif, 1996; Finnie, 1992; Hall, 2001; Harris, 1986; Morris & Klein, 2001; Rogers, 1996; Swigert, 2009; Wolf & Glass, 1992). *Sucking rhythm* is determined by the overlapping nature of SSBC innervations from the cranial nerves (trigeminal, facial, glosso-pharyngeal, vagal, accessorius, hypoglossus), cervical nerves 1–7 and thoracic nerves 1–12 (Seikel *et al.*, 2010; Wolf & Glass, 1992). The overlapping function ensures the synergetic, rhythmic and synchronous flow between sucking, swallowing and breathing in infants (Barlow, 2009; Oetter *et al.*, 1995; Wolf & Glass, 1992). Any disturbance in the innervations of one of the components of SSBC may cause arhythmic sucking, swallowing or breathing, which will be an observable indication of a disturbance in SSBC. The *cranio-cervical position* is the alignment of the head and neck with slight neck flexion that optimally opens both the oesophagus and trachea (Morris & Klein, 2001; Wolf & Glass, 1992). If a neutral cranio-cervical position is not maintained, the mobility of the hyoid bone is affected (Wolf & Glass, 1992), thereby causing a disturbance in SSBC. It is clear that *postural control, postural alignment* (including cranio-cervical position and feeding position) and *sucking rhythm* should be included in a clinical assessment.

Infantile colic is associated with an increase risk for psychosocial conditions such as postnatal depression in the mother (Vik *et al.*, 2009), poor mother-infant interaction (Brown *et al.*, 2009), sleep disturbances and tantrums in the infant (Hemmi *et al.*, 2011), infant difficulties with emotional regulation (Gomez, Baird & Jung, 2004), family strain and poor family relationships (Canivet, Jakobsson & Hagander, 2000; Räihä, Lehtonen, Huhtala, Saleva & Korvenranta, 2002). The presence of infantile colic is also associated with an increased risk for infant neglect, abuse, being shaken and death (Barr, Trent & Cross, 2006). These factors suggest a continuum of risks (Rossetti, 2001) in the infant and the family, which may influence early communication development and psychological well-being. Difficulties that impact negatively on the development of swallowing and feeding skills often contribute to educational difficulties later in life (McKirdy, Sheppard, Osborne & Payne, 2008), justifying the inclusion of infants with infantile colic in early communication intervention programmes. The presence of SSBC difficulties and the clinical assessment thereof may guide intervention and future research into the role of speech-language therapists in infants with infantile colic. 

## Aims

To explore the feeding in infants with infantile colic, the research had two aims. The first aim was to compile a clinical assessment protocol for SSBC. The second aim was to clinically assess and describe SSBC in a group of infants with colic (research group) and compare the findings with a group of infants without the condition (control group). 

## Method

Ethical clearance was obtained from the research ethics committee of the Faculty of Humanities at the University of Pretoria. All participants gave informed consent. For the first aim a literature study was conducted. For the second aim a comparative two-group research design was used to clinically observe SSBC in a group of infant participants who were independently diagnosed with the condition, in contrast with a control group without the condition. A non-randomised sample was selected of infants referred by local clinics or medical practitioners to a speech-language therapy practice in two rural towns in the North-West province of South Africa. Correlation coefficients were calculated to determine whether relationships exist between the SSBC in a group of infants with colic and those without the condition.

### Participants

A research group of 50 infant participants with colic and 28 control participants without colic were selected using a snowball selection procedure according to four age categories. The participants with infantile colic were independently diagnosed by their medical practitioners according to the Wessel *et al.*’ s (1954) definition of the condition. The selection criteria were as follows:

- The infants had to be between 1 and 17 weeks old and born at 37 weeks gestation or later, as literature indicates the condition is present in infants 0–4 months old (Savino & Tarasco, 2010). The prenatal history was required to determine the presence of risk factors.
- No risk factors such as low birth weight, poor weight gain, growth retardation, prematurity, maternal smoking, congenital anomaly or any neonatal medical conditions (allergy, reflux, gastrointestinal difficulties and esophagitis) should have been present.
- Participants should not have been using any medication, as this could influence behaviour and may have decreased the reliability of observations.
- The infants should have been cared for by the parents during the day, so that parental reporting on infantile colic would be reliable.
- The infants could be breastfed or bottle fed as literature indicates that infantile colic occurs in both breastfed and bottle fed infants (Deshpande, 2003).

The participants came from different socio-economic groups. Some participants only had access to their community clinic where fees were minimal whilst other had access to private medical services.

Table 1a–d displays the characteristics of the participants.

**TABLE 1a:** Description of participants according age category 1: 2-4 weeks (*n* = 26)

Characteristic	Research group (*n* = 19) (with infantile colic)	Control group (*n* = 7) (without infantile colic)
**Gender**		
Male	9	3
Female	10	4
**Feeding method**		
Beast feeding	7	3
Bottle feeding	8	4
Breast and Bottle	4	0
**Birth weight**		
Range	2.54–4.17 kg	2.58–3.2 kg
Average	3.3 kg	2.9 kg
**Duration of feed**		
Range	15 minutes to 60 minutes	15 minutes to 45 minutes
Average	37 minutes	21 minutes
**Frequency of feeding**		
Range	Every hour to 4 hours	Every 2½ hours to 3½ hours
Average	Every 2½ hours	Every 3 hours

**TABLE 1b:** Description of participants according age category 2: 5-8 weeks (*n* = 24)

Characteristic	Research group (*n* = 17 (with infantile colic))	Control group (*n* = 7) (without infantile colic)
**Gender**		
Male	12	5
Female	5	2
**Feeding method**		
Breast feeding	6	3
Bottle feeding	8	3
Breast and bottle	3	1
**Birth weight**		
Range	2.24–4.15 kg	2.5–3.7 kg
Average	3.2 kg	3 kg
**Duration of feed**		
Range	10 minutes to 60 minutes	15 minutes to 40 minute
Average	40 minutes	23 minutes
**Frequency of feeding**		
Range	Every hour to 4 hours	Every 2 hours to 4 hours
Average	Every 2½ hours	Every 3 hours

**TABLE 1c:** Description of participants according age category 3: 9-12 weeks (*n* = 14)

Characteristic	Research group (*n* = 7) (with infantile colic)	Control group (*n* = 7) (without infantile colic)
**Gender**		
Male	3	3
Female	4	4
**Feeding method**		
Breast feeding	0	2
Bottle feeding	6	5
Breast and bottle	1	0
**Birth weight**		
Range	2.8–3.85 kg	2.7–3.8 kg
Average	3.2 kg	3 kg
**Duration of feed**		
Range	10 minutes to more than 45 minutes	10 minutes to 20 minutes
Average	29 minutes	16 minutes
**Frequency of feeding**		
Range	Every 2 hours to 4 hours	Every 2 hours to 4 hours
Average	Every 2½ hours	Every 3 hours

**TABLE 1d:** Description of participants according age category 4: 13-19 weeks (*n* = 14)

Characteristic	Research group (*n* = 7) (with infantile colic)	Control group (*n* = 7) (without infantile colic)
**Gender**		
Male	3	3
Female	4	4
**Feeding method**		
Breast feeding	2	2
Bottle feeding	3	5
Breast and bottle	2	0
**Birth weight**		
Range	2.17–4.00 kg	2.5–3.1 kg
Average	2.9 kg	2.9 kg
**Duration of feed**		
Range	10 minutes to more than 45 minutes	10 minutes to 20 minutes
Average	29 minutes	16 minutes
**Frequency of feeding**		
Range	Every hour to 4 hours	Every 3 hours to 4 hours
Average	Every 2½ hours	Every 3 hours

According to Table 1 the participants in the two groups were fairly similar regarding gender and birth weight, but differed greatly regarding duration and frequency of feeds. Fewer participants in the research group than in the control group were breastfed.

### Material and data collection

An assessment protocol was compiled from feeding assessment forms in literature (Arvedson & Brodsky, 2002; Swigert, 2009; Wolf & Glass, 1992). Descriptions of postural control in infants were added (Alexander *et al.*, 1993; Bly, 1995). The content of the assessment protocol is described in Table 2. The final assessment protocol is included in Appendix 1.

**TABLE 2:** Content of the assessment protocol for sucking, swallowing and breathing coordination.

Area of assessment	Description
Postural control	Normal postural control develops over weeks in the 0–4-month-old infant. The postural control of a 4-week-old infant will be different from a 12-week-old infant. Alexander *et al.* (1993), Bly (1995) and Swigert (2009) were used to identify the typical postural control in every age category in the study.These authors described the developmental milestones in months. The developmental milestones were kept unchanged but the age was indicated in weeks (therefore 1 month was indicated as 4 weeks). Indicating milestones in weekly age categories eased the task of assessing each infant according to their accurate age category. Indicating the age in weeks added to the validity of the study.
Postural alignment	The description for correct postural alignment for feeding described by Alexander *et al.* (1993), Swigert (2009) and Wolf and Glass (1992) was used.
SSBR	The suck, swallow and breathing rhythm as described by Swigert (2009) and Wolf and Glass (1992) was used to for this part of the assessment.

SSBR, suck, swallow and breathing rhythm.

All participants were observed in prone, supine, supported standing and supported sitting for the appropriate postural control and alignment (Alexander *et al.*, 1993; Bly, 1995; Hall, 2001;****** Swigert, 2009) followed by eliciting nutritive sucking. Feeding by the mother was observed. The researchers have combined experience in the field of paediatric dysphagia and received training in neurodevelopmental assessment and therapy for infants, as well as neurodevelopmental care for preterm infants. A nominal value was given to absence or presence of items on the assessment protocol.

### Data analysis

Participants were divided in age categories of 2–4, 5–8, 9–12 and 13–19 weeks old. Since the number of participants in the different age categories was small, non-parametric statistics were applied to compare the components of SSBC (postural control, postural alignment and suck, swallow and breathing rhythm). The T-test with Cohen’s *d*-values was used to determine the practical significance of the differences in the duration and frequency of feeding in the research and control groups. The chi-squared test was used to determine the statistical significance of differences between the research and control groups. Cramer’s V-value was used to determine the effect size. The independent t-test and Cronbach’s alpa test were used to determine the statistical significance and internal consistency of differences found in postural alignment and SSBC between the research and control groups.

### Reliability and validity

To ensure internal validity and reliability the participants in the research group were independently diagnosed by the family’s medical practitioner and the researcher was not part of the diagnostic procedure. All participants were assessed by the same clinician and all data entries were controlled by a second person. A second observer, blind to the presence or absence of infantile colic, was used to affirm the researcher’s observations. To enhance external validity, strict selection criteria were set and the assessment protocol was conducted during a scheduled feeding time.

## Results

Table 3a–c indicates the effect size of the difference in duration and frequency of feedings in the research and control groups of each age category.

**TABLE 3a:** Effect size for the difference in duration and frequency of feedings in the research and control groups of each age category (age category 1: 2–4 weeks [*n* = 25]).

Variable	Research group (with infantile colic)		Control group (without infantile colic)		Effect size *d*-value
	Average	SD	Average	SD	
Feeding duration in minutes	37.32	15.79	22.57	11.44	0.93 Large effect
Frequency of feeding	2.63	0.70	3.18	0.31	0.78 Medium effect

SD, standard deviation.

**TABLE 3b:** Effect size for the difference in duration and frequency of feedings in the research and control groups of each age category (age category 2: 5–8 weeks [*n* = 25]).

Variable	Research group (with infantile colic)		Control group (without infantile colic)		Effect size *d*-value
	Average	SD	Average	SD	
Feeding duration in minutes	31.60	17.64	20	7.16	0.66 Medium effect
Frequency of feeding	2.68	0.75	3.14	0.63	0.62 Medium effect

SD, standard deviation.

**TABLE 3b:** Effect size for the difference in duration and frequency of feedings in the research and control groups of each age category (age category 3: 9–12 weeks [*n* = 14]).

Variable	Research group (with infantile colic)		Control group (without infantile colic)		Effect size *d*-value
	Average	SD	Average	SD	
Feeding duration in minutes	27.43	15.26	15.86	4.14	0.76 Medium effect
Frequency of feeding	2.4	0.73	3.0	0.58	0.82 Large effect

SD, standard deviation.

The differences in duration and frequency of feedings of the research and control groups indicate medium and large effect sizes and a practical significance. Feeding in participants with infantile colic took longer and was more frequent than in participants without the condition.

Table 4 provides a comparison of the results of the assessment protocol in the research and control groups in the age category 2–4 weeks.

**TABLE 3c:** Effect size for the difference in duration and frequency of feedings in the research and control groups of each age category (age category 4: 13–19 weeks [*n* = 14]).

Variable	Research group (with infantile colic)		Control group (without infantile colic)		Effect size *d*-value
	Average	SD	Average	SD	
Feeding duration in minutes	22.86	9.51	15.00	4.10	0.83 Large effect
Frequency of feeding	2.54	1.14	3.40	0.24	0.72 Medium effect

SD, standard deviation.

Almost all differences in postural control, postural alignment and suck, swallow and breathing rhythm (SSBR) between the two groups were significant (*p* < 0.05). It was only neck righting, grasp reflex, hand-to-mouth contact, pull to sit, supported standing and cup-shaped tongue that were not significant. A strong correlation between the postural control in prone and the presence of infantile colic was indicated by Cramer’s V-value. Participants with infantile colic took less weight on the shoulder girdle, had less neck extension and less hip flexion with pelvic elevation than expected for their age category on the assessment protocol. During postural adaptation for feeding the participants with colic did not assume a neutral cranio-cervical position and did not display hip flexion in one or both lower extremities. The infants with colic did not have a 1:1:1 ratio for suck, swallow and breathing or pausing between sucking cycles. Table 4 indicates a correlation between postural control, postural alignment during feeding and SSBR and the presence of colic in participants in the age category 2–4 weeks.

Table 5 provides a comparison of the research and the control groups in the age category 5–8 weeks.

**TABLE 4:** Comparison between participant groups in the category 1: 2–4 weeks (*n* = 26).

Variable	Research group (with infantile colic [%])	Control group (without infantile colic [%])	*p*-value	*V*-value
**Postural Control**				
Physiological flexion	57.89	100.00	0.04	0.38
Ventral suspension	36.84	100.00	0.004	0.49
Neck righting reaction	78.95	100.00	0.19	0.25
Rhythmic alternating movements	52.63	100.00	0.024	0.40
Arm flexion	52.63	100.00	0.024	0.40
Hand-to-hand or hand-to-mouth contact	84.21	100.00	0.26	0.21
Grasp reflex	94.74	100.00	0.54	0.12
Supported sitting	68.42	100.00	0.09	0.32
Pull to sit	100.00	100.00	1.00	0.00
Prone position	15.79	100.00	0.0001	0.61
Supported tanding	73.68	85.71	0.52	0.13
**Postural alignment**				
Cranio-cervical position	26.32	100.00	0.00	0.55
Arm flexion to midline	57.89	100.00	0.04	0.38
Slight rounded back	57.89	100.00	0.04	0.38
Hip flexion	26.32	100.00	0.00	0.55
**SSBR**				
Ratio 1:1:1	26.32	100.00	0.00	0.55
Sucking cycle present	42.11	100.00	0.01	0.46
Reduction in sucking cycle	52.63	100.00	0.02	0.40
Pauses between sucking cycle	15.79	100.00	0.00	0.61
Rhythmic feeding pattern	42.11	100.00	0.01	0.46
Lip closure reaction	47.37	100.00	0.01	0.43
Cup-shaped tongue configuration	57.89	85.71	0.19	0.25

SSBR, suck, swallow and breathing rhythm.

Statistically significant differences regarding postural control, postural alignment and SSBR were found. A correlation between the presence of colic and the absence of a neutral cranio-cervical position, quality of hip flexion and ratio of suck, swallow and breathing was found. Cramer’s V-value indicated an effect size of greater than 0.5 for the findings for aspects of the above components of SSBC. During postural adaptation for feeding the participants with colic did not assume a neutral cranio-cervical position with a slightly curved back. They did not display hip flexion in one or both lower extremities and did not have a 1:1:1 ratio for suck, swallow and breathing with poor pausing between sucking cycles.

Table 5 indicates that the correlation between postural alignment during feeding and SSBR and the presence of colic was sustained in participants in the age category 5–8 weeks.

Table 6 gives a comparison of the results of the assessment protocol for the study and the control groups in the age category 9–12 weeks.

**TABLE 5:** Comparison between participant groups in the category 2: 5–8 weeks (*n* = 24).

Variable	Research group (with infantile colic [%])	Control group (without infantile colic [%])	*p*-value	*V*-value
**Postural control**				
Ventral suspension	58.82	100.00	**0.04**	0.38
Head righting reaction	64.71	100.00	**0.07**	0.35
ATNR present	52.94	71.43	0.40	0.17
Supported sitting	41.18	100.00	**0.01**	0.48
Pull to sit	94.12	100.00	0.51	0.13
Prone position	41.18	100.00	**0.008**	0.48
Supported standing	58.82	100.00	**0.04**	0.38
**Postural alignment**				
Cranio-cervical position	23.53	85.71	**0.00**	**0.50**
Arm flexion to midline	58.82	85.71	0.20	0.25
Slight rounded back	35.29	100.00	**0.00**	**0.51**
Hip flexion	23.53	85.71	**0.00**	**0.50**
**SSBR**				
Ratio 1:1:1	23.53	100.00	**0.00**	**0.57**
Sucking cycle present	41.18	100.00	**0.01**	0.48
Reduction in sucking cycle	29.41	100.00	**0.00**	**0.54**
Pauses between sucking cycle	11.76	85.71	**0.00**	**0.58**
Rhythmic feeding pattern	23.53	100.00	**0.00**	**0.57**
Lip closure reaction	52.94	100.00	**0.03**	0.41
Cup-shaped tongue configuration	64.71	100.00	**0.07**	0.35

Again statistically significant differences regarding postural control were found between the study and control groups as well as a correlation with the presence of colic. All the aspects of postural alignment and SSBR were statistically significant with a strong correlation with the presence of colic. It appears that postural alignment and SSBR played an increased role in the presence of colic in the participants. Table 6 also indicates a correlation between postural alignment, SSBR and the presence of colic in the age category 9–12 weeks.

Table 7 gives the results in the age category 13–19 weeks.

**TABLE 6:** Comparison between participant groups in the category 3: 9–12 weeks (*n* = 14).

Variable	Research group (with infantile colic [%])	Control group (without infantile colic [%])	*p*-value	*V*-value
**Postural control**				
Ventral suspension	42.86	100.00	0.02	0.53
Head righting reaction	57.14	100.00	0.05	0.46
Midline position	0.00	100.00	0.00	0.70
Weight shift	14.29	100.00	0.00	0.65
Supported sitting	0.00	100.00	0.00	0.70
Pull to sit	71.42	100.00	0.13	0.38
Prone position	14.29	100.00	0.00	0.65
Supported standing	57.14	85.71	0.24	0.30
**Postural alignment**				
Cranio-cervical position	0.00	85.71	0.00	0.65
Arm flexion to midline	42.86	100.00	0.02	0.53
Slight rounded back	28.57	100.00	0.00	0.60
Hip flexion	28.57	85.71	0.03	0.50
**SSBR**				
Ratio 1:1:1	14.29	100.00	0.00	0.65
Sucking cycle present	14.29	100.00	0.00	0.65
Reduction in sucking cycle	0.00	100.00	0.00	0.70
Pauses between sucking cycle	14.29	100.00	0.00	0.65
Rhythmic feeding pattern	28.57	100.00	0.01	0.60
Lip closure reaction	42.86	100.00	0.02	0.53
Cup-shaped tongue configuration	14.29	85.71	0.00	0.58

SSBR, suck, swallow and breathing rhythm.

Once again statistically significant differences were indicated between postural control, postural alignment and SSBR of the study and control groups with a correlation between the presence of colic and the quality of postural control. Cramer’s V-values indicate that the postural alignment and SSBR play an increasing role in the presence of colic. Participants with colic had difficulty playing with hands to knees when in the supine position and rolling to the side. All participants in the control group were able to do so. The research group had difficulty pushing up on their elbows and shifting weight with the shoulder girdle. The research group also had difficulty with accidental rolling, stood with a wide base of support, had poor quality of supported sitting and showed difficulty when pulled to sit. Again, Table 7 indicates an even stronger correlation between postural alignment, SSBR and the presence of colic in the age category 13–19 weeks.

The results, of all four age categories, indicate a large effect size and a correlation with the presence of infantile colic.

Table 8 displays the results of a t-test and a Cronbach’s alpha validity coefficient.

**TABLE 7:** Comparison between participant groups in the category 4: 13–19 weeks (*n* = 14).

Variable	Research group (with infantile colic [%])	Control group (without infantile colic [%])	*p*-value	*V*-value
**Postural control**				
Ventral suspension	100.00	100.00	1.00	0.00
Supine	0.00	100.00	0.00	0.71
Supported sitting	14.29	100.00	0.00	0.65
Pull to sit	42.86	100.00	0.02	0.53
Prone position	0.00	100.00	0.00	0.70
Supported standing	14.29	85.71	0.00	0.58
**Postural alignment**				
Cranio-cervical position	14.29	100.00	0.00	0.65
Arm flexion to midline	28.57	100.00	0.00	0.60
Slight rounded back	0.00	100.00	0.00	0.70
Hip flexion	28.57	85.71	0.03	0.50
**SSBR**				
Ratio 1:1:1	28.57	100.00	0.00	0.60
Sucking cycle present	57.14	100.00	0.05	0.46
Reduction in sucking cycle	57.14	100.00	0.05	0.46
Pauses between sucking cycle	28.57	85.71	0.03	0.50
Rhythmic feeding pattern	28.57	100.00	0.00	0.60
Lip closure reaction	28.57	100.00	0.00	0.60
Cup-shaped tongue configuration	28.57	85.71	0.03	0.50

The results of the t-test indicate a statistically significant difference between the postural alignment and SSBR of all participants with and without colic. The Cronbach’s alpha value indicates a good internal reliability for postural alignment across age categories.

It is commonly accepted that poor postural control negatively impacts on postural alignment and disturbs feeding and swallowing (Hall, 2001; Redstone & West, 2004). This pattern is well-documented in infants with neurological difficulties (Sheppard, 2008) but not in infants with colic. Table 7 and Table 8 indicate this same pattern of poor postural control with a negative impact on postural alignment resulting in a feeding disturbance.

**TABLE 8:** T-test results for postural alignment and SSBR in both participant groups.

Section of Assessment protocol	Research group (with infantile colic)		Control group (without infantile colic)		*p*-value	Number of items	*α* value
	Average	SD	Average	SD			
Postural alignment present	1.36	1.08	3.79	0.42	**0.0000**	4	**0.86**
SSBR present	2.46	1.80	6.82	0.39	**0.0000**	7	**0.74**

SSBR, suck, swallow and breathing rhythm.

In the age category 2–4 weeks, five descriptors for postural control were found to be not significant (neck righting reaction, hand-to-hand or hand-to-mouth contact, grasp reflex, pull to sit and supported standing). In the age category 5–8 weeks, three descriptors for postural control and postural alignment were found to be not significant (presence of asymmetric tonic neck reflex, pull to sit and arm flexion to midline). In the age category 9–12 weeks, two descriptors for postural control were found to be not significant (supported sitting and supported standing). In the age category 13–19 weeks, only one descriptor for postural control was found to be not significant (ventral suspension).

## Discussion

The main purpose of this study was to explore the SSBC in young infants with infantile colic.

The participants with infantile colic took longer than the normal 20 minutes or less (Arvedson & Brodsky, 2002) to complete a feeding. They also fed more frequently with less than three hours between feeds. This may offer an explanation for the perception in general and in popular literature that infantile colic is associated with feeding difficulties. The finding also strengthens the rationale for exploring feeding difficulties in infants with infantile colic.

An evaluation protocol for SSBC was compiled to clinically assess and compare a group of infants with and without the condition. The results indicate that SSBC can be assessed clinically and the assessment protocol could now be included in assessment and treatment planning for infants with colic.

The results indicated that postural alignment and SSBR of participants with colic differed significantly from participants without the condition across age categories. The difficulties with postural control, postural alignment and SSBC appear to be subtle and present as feeding difficulty or infantile colic. Redstone and West (2004) also indicate a correlation between the quality of postural alignment and the quality of feeding. The results highlight the importance of clinically assessing SSBC in infants with infantile colic in order to inform and influence clinical practice. The results are in agreement with Miller-Loncar *et al.* (2004), who also suggest that feeding difficulties are associated with infantile colic.

The components of SSBC not statistically significant between the groups strengthen the importance of assessing SSBC clinically in infants with the condition. Poor postural control and a negative impact on SSBC is found in infants with neurological difficulties (Arvedson & Lefton-Greif, 1996; Hall, 2001; Lefton-Greif & McGrath-Morrow, 2007; Wolf & Glass, 1992). Although participants in the present study did not have neurological difficulties, a similar pattern emerged in the present study. With an increase in age, increasingly more aspects of postural control differed significantly between the research and control groups. Literature indicates that infants develop more muscle control as reflexes diminish, enabling better postural adaptations for feeding (Alexander *et al.*, 1993; Bly, 1995; Redstone & West, 2004).

The pattern of poor postural control impacted similarly, but in a subtle way, on postural alignment, resulting in a disturbance of SSBC. The feeding disturbance is much more subtle than in infants with neurological difficulties and may present as the symptoms parents describe for infantile colic. The findings suggest some truth in the perception that infantile colic is the result of feeding difficulties.

Infantile colic is further associated with the occurrence of communication-interaction difficulties between parent and infant, serious psychosocial difficulties, abuse and educational difficulties, which suggest a continuum of risk in infants with the condition. The results suggest the importance of a clinical assessment of SSBC and the involvement of a speech-language therapist for early feeding and communication intervention.

Although there appears to be some evidence of subtle disturbances in SSBC associated with infantile colic, the causes of colic still need to be investigated. Due to the small sample size the findings of this study cannot be generalised. Since a non-randomised convenient sampling method involving only two communities was used, bias may be present in the sample. It is recommended that further research should make use of larger sample sizes and involve more communities. 

## Conclusion

A need was identified to explore the importance of including SSBC as a possible contributing factor to infantile colic. Considering the high prevalence (10%–40%) of infantile colic (Deshpande, 2003), and by implication the risk for communication development delays, this article suggests that assessment of SSBC should be included in the diagnosis of infants with colic. Literature provides well-documented treatment options for the components of SSBC (Arvedson & Brodsky, 2002; Wolf & Glass, 1992), which may improve the outcome of the condition in very young infants. Speech-language therapists play an important role in the identification, intervention and outcome of feeding difficulties in young infants (ASHA, 2008). Further research on the topic may expand the role of the speech-language therapists in early intervention. 

## Appendix A

### Assessment protocol for suck, swallow and breathing coordination (SSBC)

#### 1. Postural control

##### 1–4 weeks

**Table:**

Yes	No	Postural control
		Physiological flexion
		Total flexion in ventral suspension
		Neck righting reaction
		Rhythmic alternating movement of extremities, lifted off the surface
		Arms in flexion close to the body
		Positional hand-to-hand contact and hand-to-mouth contact in side lying
		Strong grasp reflex
		Supported sitting: Slightly rounded back, head falls to the front, scapulas in abduction
		Pulls to sit: Head lag
		Prone: Able to turn head to the side, hip flexion and pelvic elevation, weight predominantly on shoulder girdle, lifts head with neck extension
		Supported standing: Automatic stepping, elbow extension, attempts to lift head

##### 5–8 weeks

**Table:**

Yes	No	Postural control
		Leg flexion in ventral suspension and head horizontal with back
		Head righting reaction when infant is tilted forward and backwards
		Asymmetric tonic neck reflex present
		Supported sitting: Slightly rounded back, head bouncing
		Pulls to sit: Head lag
		Prone: Lift head 45°, slight weight shifts in the body towards the same side
		Supported standing: No support of weight on feet

##### 9–12 weeks

**Table:**

Yes	No	Postural control
		Leg flexion in ventral suspension and head horizontal with back
		Head righting reaction in all positions
		Maintain midline positions
		Lateral weight shifts through head, shoulders and trunk
		Supported sitting: Sits with increased moments of head control
		Pulls to sit: Head lag followed by head lift close to sitting position
		Prone: Lifts head and hold position
		Supported standing: Support weight through feet with a wide base

##### 13–19 weeks

**Table:**

Yes	No	Postural control
		Legs and head horizontal with back in ventral suspension
		Supine: Plays with hands to knees, rolls to side
		Supported sitting: Sit with scapula adduction, shoulder elevation and arm abduction
		Pull to sit: Pulls chin towards chest, head in midline and actively assists with pull
		Prone: Push up on elbows, weight shifts through shoulder girdle. Accidental rolling
		Standing: Support weight with feet, strong standing with wide base

*Source*: Alexander *et al.* (1993); Bly (1995); Hall (2001)

#### 2. Postural alignment for feeding

**Table:**

Yes	No	Components of postural alignment
		Neutral cranio-cervical position
		Flexion of the arms in direction of the vertical midline
		Slightly rounded back
		Slight hip flexion in one or both of the lower extremities

*Source*: Alexander *et al.* (1993); Swigert (1998); Wolf & Glass (1992).

#### 3. Suck, swallow and breathing rhythm (SSBR)

**Table:**

Yes	No	Components of suck, swallow and breathing rhythm
		Ratio of suck, swallow and breathing = 1:1:1
		Duration of initial sucking cycles 20–30 seconds
		Pattern of gradual decrease in sucking cycles
		Pauses of 5 seconds between sucking cycles
		Maintains a rhythmic feeding pattern
		Lip closure reaction when nipple or bottle teat enters the mouth
		Cup-shaped tongue configuration when nipple or bottle teat is offered

*Source*: Swigert (1998); Wolf and Glass (1992).
